# Prevalence of Parkinsonism Among Foundry Workers in an Automobile Manufacturing Factory in Tehran

**DOI:** 10.7759/cureus.28685

**Published:** 2022-09-01

**Authors:** Mohammad Rohani, Negin Kassiri, Maziar Emamikhah Abarghouei, Saber Mohammadi, Yasser Labbafinejad

**Affiliations:** 1 Skull Base Research Center, Iran University of Medical Sciences, Tehran, IRN; 2 Department of Neurology, School of Medicine, Iran University of Medical Sciences, Tehran, IRN; 3 Department of Occupational Medicine, School of Medicine, Iran University of Medical Sciences, Tehran, IRN; 4 Occupational Medicine Research Center, Department of Occupational Medicine, School of Medicine, Iran University of Medical Sciences, Tehran, IRN

**Keywords:** foundry, prevalence, manganese, transcranial sonography, neurological examination, parkinsonism

## Abstract

Background

Manganese, as an essential element, has neurotoxic effects on basal ganglia and causes parkinsonism, dystonia, and cognitive symptoms in exposed individuals. Transcranial sonography (TCS) is a noninvasive and easily accessible imaging modality for detecting the accumulation of trace elements in the basal ganglia.

Methodology

In a cross-sectional study of foundry workers of one of the automobile manufacturing companies in 2019, the prevalence of parkinsonism was assessed through neurological examination and brain parenchymal sonography or TCS. The prevalence of parkinsonism according to age, smoking, work experience, marital status, and exposure to manganese was determined.

Results

Among 83 male workers, the prevalence of parkinsonism according to neurological examination, substantia nigra hyperechogenicity on TCS, lentiform nucleus hyperechogenicity, and totally was 33.7%, 9.6%, 10.8%, and 42.2%, respectively. The association between the prevalence of parkinsonism and age, smoking, work experience, marital status, and manganese exposure was evaluated. Parkinsonism according to lentiform nucleus hyperechogenicity was associated with smoking (odds ratio [OR] (95% confidence interval [CI]) = 26.63 (2.38-178.71)) and work experience (OR (95% CI) = 7.18 (0.84-61.32)).

Conclusions

According to this study, the prevalence of parkinsonism based on neurological examination or brain sonography findings was 42.2%. The implementation of this combined screening method might facilitate earlier detection of affected individuals among manganese-exposed workers.

## Introduction

Manganese is an essential element that contributes to many cellular metabolic mechanisms and protects against lipid peroxidation. This metallic element has extensive usage in producing metal alloys, especially stainless steel. It is also being used in dry battery manufacturing, color, and pesticide production [1]. It has neurotoxic effects on basal ganglia, disturbs mitochondrial oxidative metabolism, and debilitates anti-oxidative cellular defense. Clinically, toxic levels of manganese can cause parkinsonism, dystonia, and cognitive symptoms in exposed individuals [1,2]. Several studies have shown that manganese exposure even at lower levels than the permissible level can be associated with parkinsonism and neuropsychiatric dysfunction [3-5].

Transcranial sonography (TCS) is one of the brain imaging modalities which can be used for detecting the accumulation of trace elements in basal ganglia [6,7]. The advantages of this noninvasive method are the possibility of providing high-resolution images of deep brain tissues with low costs and easy accessibility. However, TCS has certain limitations including the poor temporal bone window in some cases and dependency on operator experience and skill [8,9].

Studies have reported controversial findings on the association between manganese exposure and parkinsonism [10-18]. In this study, we assessed the association between manganese exposure and parkinsonism in foundry workers and the prevalence of parkinsonism based on neurological examination and transcranial brain sonography.

## Materials and methods

We designed a cross-sectional study to determine the prevalence of parkinsonism among foundry workers in an automobile manufacturing factory in Tehran in 2019. All 83 male employees who were exposed to manganese fumes from different work units, including the production line, forklift driving, quality control, melt, power, and furnace mechanics, participated in the study. First, demographic and occupational data such as age, work experience, smoking, education, marital status, work units, and shift working were collected through a questionnaire. The level of manganese exposure in different work units was determined by occupational hygiene experts and approved by health, safety, and environment experts. Participants were categorized into two groups, namely, low exposure (production line, forklift driver, quality control) and high exposure (melt, power, furnace mechanics). Then, for all participants, neurological examinations to detect hypokinesia, rigidity, tremor, and postural instability were performed by a movement disorders neurologist. Cases with hypokinesia and one other sign (tremor, rigidity, and postural instability) were considered clinically parkinsonism [19]. Subsequently, TCS was conducted for all participants, with a Sonos 5500 ultrasound system (Philips, Amsterdam, the Netherlands) equipped with a 2-2.5 MHz probe through a temporal window evaluating the echogenicity of the substantia nigra (on the same side) and the lentiform nucleus (opposite side). For the echogenicity of the substantia nigra, the cut-off point was ≥0.2 cm², and regarding the lentiform nucleus, every increase in signal was considered abnormal. Participants with work experience of fewer than two months were excluded. The data were analyzed by SPSS version 24 (IBM Corp., Armonk, NY, USA). Logistic regression analysis was used to adjust confounding variables. All variables with p-values of <0.05 were considered significant. The prevalence of parkinsonism according to age, smoking, work experience, education, marital status, shift work, and work units was determined. Before starting the study, verbal informed consent was taken from all participants. This study was approved by the Research Ethics Committees of the School of Medicine of Iran University of Medical Sciences (approval number: IR.IUMS.FMD.RC.1398.198, obtained on August 25, 2019).

## Results

In total, 83 male workers were included, with a mean average age of 38.44 ± 7.17 years. The minimum age was 22, and the maximum was 57. Overall, 12% of workers were single, and 88% were married. Most of the workers had a high school education (78.3%). Additionally, 31.3% were smokers with an average smoking history of 9.44 packs/year. The mean environmental manganese exposure was 3.34 mg/m³, which was below the permissible exposure limit (5 mg/m³). Overall, 83.1% had rotational shift work (one week from 6 AM to 6 PM and one week from 6 PM to 6 AM). Mean work experience was 12.3 years, with a minimum of six months and a maximum of 23 years. The abnormal neurological findings and TCS findings are illustrated in Table 1.

**Table 1:** Neurological examination and TCS findings. TCS: transcranial sonography

Neurological examination	N (%)	Increased signal of lentiform nucleus	N (%)	Right substantia nigra size (cm^2^)	N (%)	Left substantia nigra size (cm^2^)	N (%)	Both sides of substantia nigra size (cm^2^)	N (%)
Hypokinesia +1	26 (31.3%)	Right side	7 (8.4%)	0.00	71 (85.5%)	0.00	69 (83.1%)	0.00	77 (92.7%)
Hypokinesia +2	2 (2.4%)	Left side	6 (7.2%)	0.01–0.19	7 (8.4%)	0.01–0.19	8 (9.6%)	0.01–0.19	4 (4.81%)
Rigidity	6 (7.2%)	Both sides	5 (6.0%)	≥0.20	5 (6.0%)	≥0.20	6 (7.2%)	≥0.20	2 (2.4%)
Decreased arm swing	2 (2.4%)			Total	83 (100%)	Total	83 (100%)	Total	83 (100%)

The prevalence of parkinsonism was assessed according to the following rationales: (1) substantia nigra echogenicity area ≥0.2 cm² on at least one side (prevalence: 10.8%). (2) Hyperechogenicity of the lentiform nucleus on at least one side (prevalence: 9.6%). (3) Positive findings on TCS (either substantia nigra size or hyperechogenicity of lentiform nucleus) (prevalence: 18.1%). (4) Positive findings on neurological examination (prevalence: 33.7%). (5) Positive findings on at least one of the above (prevalence: 42.2%). The prevalence of parkinsonism according to the five explained patterns and the association with other variables are presented in Table 2.

**Table 2:** Prevalence of parkinsonism according to different defined patterns and association with other variables. *Including production line, forklift driver, and quality control. **Including melt, power, and furnace mechanics. ***All workers with substantia nigra size ≥ 0.2 cm² on the left (6 patients) or right (5 patients) or both sides (2 patients) were included. Based on our data 2 workers had both sides involved; therefore, the final report (9 patients) is shown lower than the mathematic summation. ****All workers with increased signal on the left (six patients), right (seven patients), or both sides (five patients) were included. Based on our data, five workers had both sides involved; therefore, the final report (eight patients) is shown lower than the mathematic summation. *****All workers with substantia nigra size ≥0.2 cm² (nine patients), an increased signal in the lentiform nucleus (eight patients), or both of them (two patients) were included. Based on our data, two workers had both types of involvement; therefore, the final report (15 patients) is shown lower than the mathematic summation. OR: odds ratio; CI: confidence interval

		Prevalence of parkinsonism based on hyperechogenicity of the substantia nigra***, n = 9 (10.8%)	Prevalence of parkinsonism based on hyperechogenicity of the lentiform nucleus****, n = 8 (9.6%)	Prevalence of parkinsonism based on positive findings on sonography*****, n = 15 (18.1%)	Prevalence of parkinsonism based on abnormal neurologic physical examination, n = 28 (33.7%)	Total prevalence of parkinsonism, n = 35 (42.2%)
Low exposure*, N = 42 (50.6%)		2 (4.8%)	2 (2.4%)	3 (7.1%)	13 (31.0%)	15 (25.7%)
High exposure**, N = 41 (49.3%)		7 (17.1%)	6 (14.6%)	12 (29.3%)	15 (36.6%)	20 (48.8%)
	P-value	0.07	0.12	0.009	0.58	0.22
	OR (95% CI)	4.11 (0.80-21.15)	3.42 (0.65-18.09)	5.37 (1.39-20.82)	1.28 (0.51-5.20)	1.71 (0.71-4.13)
Age ≤40 years, N = 42 (50.6%)		6 (14.3%)	3 (7.1%)	7 (16.7%)	12 (28.6%)	17 (40.5%)
Age ≥40 years, N = 41 (49.3%)		3 (7.3%)	5 (12.2%)	8 (19.5%)	16 (39.0%)	18 (43.9%)
	P-value	0.30	0.43	0.73	0.31	0.75
	OR (95% CI)	0.47 (0.11-2.03)	1.80 (0.40-8.10)	1.21 (0.39-3.71)	1.60 (0.63-4.00)	1.15 (0.48-2.75)
Work experience of less than 14 years, N = 39 (46.9%)		4 (10.3%)	1 (2.6%)	5 (12.8%)	13 (33.3%)	17 (43.6%)
Work experience of more than 14 years, N = 44 (53%)		5 (11.4%)	7 (15.9%)	10 (22.7%)	15 (34.1%)	18 (40.9%)
	P-value	0.87	0.04	0.24	0.94	0.80
	OR (95%CI)	1.12 (0.27-4.51)	7.18 (0.84-61.32)	2.00 (0.61-6.47)	1.03 (0.41-2.57)	0.89 (0.37-2.14)
Negative smoking history, N = 57 (68.6%)		5 (8.8%)	1 (1.8%)	6 (10.5%)	19 (33.3%)	21 (36.8%)
Positive smoking history, N = 26 (31.3%)		4 (15.4%)	7 (26.9%)	9 (34.6%)	9 (34.6%)	14 (53.8%)
	P-value	0.36	0.000	0.008	0.90	0.14
	OR (95% CI)	1.89 (0.46-7.71)	26.63 (2.38-178.71)	4.50 (1.39-14.49)	1.05 (0.39-2.81)	2.00 (0.78-5.12)
Single, N = 10 (12%)		2 (20%)	0 (0%)	2 (20.0%)	3 (30.0%)	4 (40.0%)
Married, N = 10 (88%)		7 (9.6%)	8 (11.0%)	13 (17.8%)	25 (34.4)	31 (42.5%)
	P-value	0.32	0.27	0.86	0.79	0.88
	OR (95% CI)	0.42 (0.07-2.40)	0.86 (0.79-0.94)	0.86 (0.16-4.56)	1.21 (0.28-5.11)	1.10 (0.28-4.26)
Constant shift work, N = 14 (16.8%)		0 (0%)	1 (7.1%)	1 (7.1%)	2 (14.3%)	4 (40.0%)
Rotational shift work, N = 69 (83.1%)		9 (13.0%)	7 (10.1%)	14 (20.3%)	26 (37.7%)	31 (42.5%)
	P-value	0.15	0.72	0.24	0.09	0.85
	OR (95% CI)	0.15 (1.05-1.26)	1.46 (0.16-12.97)	3.30 (0.39-27.48)	3.62 (0.75-17.51)	3.17 (0.81-12.37)

Univariant logistic regression analysis for smoking history and exposure with the prevalence of parkinsonism based on positive findings on sonography are presented in Table 3.

**Table 3:** Univariant logistic regression analysis for smoking history and exposure with the prevalence of parkinsonism based on positive findings on sonography.

	β coefficient	Standard error	P-value	Odds ratio	Confidence interval
Exposure to manganese	-1.83	0.72	0.012	0.15	0.38-0.66
Cigarette smoking	-1.66	0.64	0.010	0.18	0.05-0.66
Constant	0.14	1.32	0.000	0.008	

## Discussion

This study aimed to investigate the association between manganese exposure and parkinsonism. This study suggests that exposure to manganese in foundry workers significantly increases the risk of parkinsonism. This finding is in contrast to studies that declined this association. In a case-control study by Goldman et al., no relationship between welding exposure and parkinsonism was found [10]. In cohort studies designed by Fored et al. and Fryzek et al., the results were similar [11,12]. Mortimer et al. designed a study to examine the associations of welding and manganese exposure with parkinsonism using meta-analyses of data from cohort, case-control, and mortality studies. They concluded that welding and manganese exposure is not associated with increased parkinsonism risk [13].

On the other hand, several studies have shown that manganese exposure increases parkinsonism prevalence in welder populations. They estimated parkinsonism prevalence of about 15% based on the Unified Parkinson’s Disease Rating Score Part III (UPDRS3) [14-17]. On secondary analysis of data from 418 South African manganese mine workers, the prevalence of parkinsonism as the primary outcome, defined by UPDRS3 ≥15, was 29.4% [18]. In this study, the prevalence of parkinsonism based on combined neurological examination and TCS findings was 42.2%, which is higher than the findings reported in previous studies [14-18]. This may be the result of our more sensitive approach combining neurological examination and brain sonography as a method for determining the prevalence of parkinsonism. A higher level of exposure in foundry workers is another possibility.

On logistic regression analysis, the prevalence of parkinsonism based on the hyperechogenicity of lentiform nucleus and TCS overall was associated with smoking history. This is in concordance with some previous studies [18]. In some studies, smoking has been negatively associated with parkinsonism [12,20].

On average, workers were exposed to an estimated 3.34 mg/m^3^ of cumulative manganese with a mean average of 12.30 years of work experience. Dlamini et al. [18] reported an estimated 3.7 mg/m^3^ of cumulative manganese with a mean average of 13.5 years of work experience. Despite the lower exposure level in this study, the prevalence of parkinsonism was higher (42.2% vs. 29.4%). This may further highlight the effect of our more sensitive approach as a screening method for parkinsonism.

In the group with a higher level of exposure, the prevalence of parkinsonism was higher. This association was statistically significant between the prevalence of parkinsonism based on TCS overall and a higher level of exposure.

The advantage of this study was a combined screening method using neurological examination and TCS performed by an expert as a screening test which led to the higher detection rates of parkinsonism among manganese-exposed workers. The limitation of the study was the impossibility of confirming the cases found by TCS with magnetic resonance imaging. Additionally, the cooperation of patients to participate in the study was reduced due to the outbreak of coronavirus disease 2019. Using the gold standard test to confirm the diagnosis of cases found by TCS and improve sample size are recommended for future studies.

## Conclusions

According to this study, the prevalence of parkinsonism based on neurological examination or brain sonography findings was 42.2%, which is higher than the previous studies. Differences in methodologies can explain this disparity. Accurate neurological examinations and additional studies such as TCS, performed by an expert, led to earlier detection of parkinsonism in workers exposed to manganese. Considering the high level of exposure in young foundry workers, which can lead to disabling conditions such as dystonia and parkinsonism, screening programs, including neurological examinations and TCS, would be helpful to improve the quality of life and prevent catastrophic movement disorders in young productive workers.
